# Perceived Barriers to NGS-Based Molecular Profiling Among US Metastatic Breast Cancer Patients

**DOI:** 10.3390/diagnostics15202626

**Published:** 2025-10-17

**Authors:** Nicholas Cadirov, Moumita Chaki, Olivia Foroughi, Omar Perez, Stella Redpath, Gary Gustavsen

**Affiliations:** 1 Health Advances LLC, Newton, MA 02466, USA; 2 AstraZeneca, Gaithersburg, MD 20878, USA

**Keywords:** next generation sequencing, metastatic breast cancer, healthcare professionals, patients, payers, personalized medicine, precision oncology, survey, genomic

## Abstract

**Background/Objectives**: Next-generation sequencing (NGS)-based molecular profiling has revolutionized personalized medicine and unlocked new treatment options for cancer patients. Clinical guideline bodies agree that patients diagnosed with HR+/HER2− metastatic breast cancer (mBC) may benefit from comprehensive somatic genomic profiling to identify candidates for established targeted therapies and clinical trials, yet many patients are not receiving it due to a lack of widespread access to NGS. **Methods**: To better understand the perceived barriers (if any) to NGS tests in mBC, a study was conducted across multiple stakeholders including medical oncologists, nurses, physician assistants, lab directors, pathologists, payers, and patients. **Results**: This study revealed that despite the awareness and recognition of the value proposition of NGS-based molecular profiling in mBC, inconsistent payer coverage, high out of pocket costs for patients, and challenges in managing reimbursement and prior authorization processes can lead to suboptimal utilization of NGS, which can subsequently lead to suboptimal treatment decisions where approved therapies exist. Interestingly, many payers (33%) were not aware of the current somatic biomarker testing recommendations from NCCN guidelines. As a result, payers identified the lack of clear clinical guidelines (74% ranked as top 3), the lack of internal consensus on which NGS tests to cover (45%), and the absence of internal expertise on NGS (39%) as the primary hurdles for broader NGS access. **Conclusions**: The results suggest that widespread HCP and payer education on clinical guidelines (e.g., NCCN) and utility for targeted therapy selection is crucial for enhanced adoption of NGS-based molecular profiling in mBC.

## 1. Introduction

The introduction of next-generation sequencing (NGS) has unlocked a new era of possibility within personalized medicine by providing comprehensive diagnostic information on patients [1]. NGS-based molecular profiling is a crucial component in cancer care today that allows clinicians to identify cancer genomic alterations, enabling informed treatment recommendations based on the tumor-specific biomarker status [2,3,4,5,6,7]. Along with identifying patients eligible for FDA-approved targeted therapies, NGS-based molecular profiling may also identify eligible patients for clinical trials based on certain biomarkers (currently unactionable) as well as the therapies that will have limited clinical benefit based on resistance mutations found in test results [8,9,10]. Several studies have demonstrated clear benefits of NGS-based molecular profiling, e.g., patients diagnosed with metastatic breast cancer (mBC) who received NGS testing and therefore the right targeted therapy for their tumors had prolonged progression-free survival (PFS) compared to the patients who did not receive NGS testing [11,12]. This holds particularly true for mBC when selecting from tier I/II alterations, as classified by the ESMO Scale for Clinical Actionability of Molecular Targets (ESCAT) [13].

In acknowledgement of the clinical utility of comprehensive testing in patients diagnosed with mBC, the National Comprehensive Cancer Network (NCCN) recommends comprehensive germline and somatic profiling to identify candidates for targeted therapies such as those targeting *BRCA1/2*, *PIK3CA*, *AKT1*, *PTEN* alterations, *ESR1*, *NTRK*, MSI-H/dMMR, TMB-H, and *RET*-fusions [14]. The guideline suggests that clinicians should test at least upon the first recurrence of disease and consider an additional test after progression to ensure that healthcare professionals are armed with the most up-to-date information [14]. Also, for a biopsy negative for actionable biomarkers, consideration of testing with an alternative specimen is recommended to ensure that clinicians have the greatest amount of information possible [14]. Similarly, the American Society of Clinical Oncology (ASCO) recommends that patients diagnosed with metastatic or advanced cancers should receive somatic genomic sequencing at diagnosis and progression, particularly via multigene panel-based assays if more than one biomarker linked therapy is approved for a patient’s disease [15]. Of note, the European Society for Medical Oncology (ESMO) Precision Medicine Working Group also advocates for NGS-based molecular profiling as a routine clinical practice in patients with advanced cancers, with the recent inclusion of advanced breast cancers (aBC) in 2024 [16].

While most medical oncologists in the United States follow clinical and society guidelines and order NGS-based molecular profiling for their patients diagnosed with mBC, not all patients are tested at the appropriate time or receive the ideal number of tests or appropriate types of NGS tests. For example, some mBC patients may receive only one NGS test during their patient journey rather than receiving multiple NGS-based molecular profiling tests to continuously identify genomic alterations reflecting their disease status over time. At times, some mBC patients may only receive one liquid-based NGS test and not a follow-up tissue-based or liquid-based NGS test when it is necessary to confirm the biomarker status. This leads to suboptimal testing as not all liquid-based results in mBC are equivalent to tissue-based NGS results [17,18]. Some clinicians may be hesitant to broadly adopt NGS-based molecular profiling due to the potential risk of sample insufficiency leading to test failure. However, significant advancements in tumor DNA extraction and the increasing utility of liquid-based testing have assuaged some fears around this [9]. Patients may sometimes not receive NGS-based molecular profiling immediately after metastatic diagnosis, but rather after lines of systemic therapies when the probability of survival has declined. This practice may hinder the opportunity for extended patient survival with a targeted therapy applied earlier in the patient journey [19]. Further, this practice contradicts clinical guideline recommendations (e.g., NCCN) and limits access to critical information required to inform therapy selections at the right time [14,15,16].

While the perceived utility of NGS-based tumor profiling and guideline recommendations have corresponded with an increase in NGS-based tumor profiling in mBC across settings [9] and the NGS testing rates are relatively high and increasing in mBC, there is still an opportunity to improve the patientcare via optimal utilization of NGS testing. To better understand the areas of improvement, a multi-stakeholder survey was conducted in the United States that includes medical oncologists, nurses, physician assistants, pathologists, lab directors, payers, and patients. To our knowledge, this study is the first of its kind to review a range of stakeholder opinions to uncover the remaining barriers to wider adoption of NGS-based molecular profiling in mBC, thereby potentially boosting the patient survival via targeted therapies.

## 2. Materials and Methods

### 2.1. Survey Methodology

Four individual quantitative online surveys with United States-based medical oncologists (*n* = 109), nurses and physician assistants (*n* = 50), lab directors and pathologists (*n* = 40), payers (*n* = 31), as well as patients diagnosed with mBC (*n* = 137) were conducted. Respondent demographics are displayed in Table 1.

### 2.2. Survey Design and Validation

A series of 60 min double blinded phone-based interviews were performed to inform development of the quantitative survey with medical oncologists, nurses, physician assistants, lab directors, pathologists, and payers. Questions were asked regarding reimbursement, coverage, tissue sample sufficiency, turnaround time, and NGS testing behavior. Survey beta-testing with representative stakeholders was also performed to ensure appropriate question and answer options, question clarity, neutrally framed questions, and overall survey flow.

To minimize selection bias, invitations were distributed across diverse clinical settings, clinical laboratories, payer types, and US geographies and stratified with quotas in place to limit oversampling of any individual demographic and ensure diversity. Multiple recruitment channels were utilized to diversify respondents, including 2 major market research vendors supplemented with an internally developed database of Health Advances experts that includes over 20,000 healthcare professionals. The survey was also designed to be fully anonymous to reduce barriers to participation and social desirability bias, encouraging honest and complete responses.

### 2.3. Study Participant Selection Criteria

To ensure high quality market research, the 367 survey respondents were recruited by market research vendors in compliance with industry standards, as well as supplemented with an internally developed database of Health Advances experts. All non-patient respondents were 21 or older at the time of the survey, while patient respondents were 18 or older at the time of the survey. Ethical review and approval were waived for this study by Advarra CIRBI Platform, using the Department of Health and Human Services regulations found at 45 CFR 46.104(d)(2), and the IRB determined that the research project is exempt from IRB oversight. All survey respondents reported involvement with tumor molecular testing either as a healthcare professional, a lab director performing NGS-based tumor profiling, a payer setting policies for reimbursement of oncology tests, or a patient diagnosed with HR+/HER2− mBC eligible for comprehensive testing.

#### 2.3.1. Medical Oncologists, Nurses, and Physician Assistants

Healthcare professional respondents (HCPs) were required to have been in practice between 2 and 40 years, spend more than 25% of their time in direct patient care, and see on average ten or more patients with HR+/HER2− metastatic breast cancer per month. Medical oncologists were required to be board certified in medical oncology.

#### 2.3.2. Lab Directors and Pathologists

Respondents were required to work in a clinical laboratory, including either an independent reference lab, academic medical center lab, community hospital lab, or academic affiliated community hospital lab, and serve either as a laboratory director, manager, or staff pathologist. Experts needed to be involved in or responsible for managing laboratory workflows or reviewing and releasing test results. Respondents had to oversee molecular testing and needed to process more than five HR+/HER2− metastatic breast cancer specimens per month.

#### 2.3.3. Payers

Respondents were required to have the title of Medical Director, Clinical Advisor, Chief Medical Officer, or Laboratory Benefits Manager with experience working at payer organizations for more than two years at a plan covering more than 5000 lives. Respondents were required to be involved in policy decision-making, either by frequently being involved (23% of respondents) or directly involved (77% of respondents) in medical policy and reimbursement decisions for oncology diagnostic tests.

#### 2.3.4. Patients

Respondents were required to have been diagnosed with metastatic HR+/HER2− (72%) or HR+/HER2 low (28%) breast cancer.

## 3. Results

### 3.1. Current NGS Testing Landscape for Patients with mBC

The majority of HCPs are aligned with clinical guidelines and the broad base of literature supporting the use of NGS-based molecular profiling at diagnosis and progression as a tool to guide therapy selection as part of a standard treatment protocol for patients diagnosed with metastatic breast cancer (Figures S1 and S2). Despite a strong rationale, HCPs report testing 70% of their eligible patients today, leaving 30% of eligible patients without comprehensive NGS panel results after metastatic diagnosis that could be used to guide targeted therapy selection and improve patient outcomes.

For most patients diagnosed with metastatic breast cancer, HCPs rely upon NGS-based molecular profiling to assess the unique genomic signature of metastatic breast tumors with either a small, targeted panel or a comprehensive panel of hundreds of genes. The small, targeted panels are commonly designed to capture a combination of actionable biomarkers from the genes including *ESR1*, *PIK3CA*, *AKT1*, and *PTEN* that can directly guide treatment today and can include tests that are both approved as companion diagnostics (CDx) as well as laboratory developed tests (LDTs). The comprehensive panels additionally include other genes that are not associated with targeted therapies today but may allow for inclusion in clinical trials of studied drugs or provide useful information for medical oncologists.

For patients diagnosed with metastatic breast cancer receiving NGS-based molecular profiling today, testing is largely performed by a send-out lab, as many health systems lack the infrastructure and capability to perform NGS testing on site (Figure S3). Lab directors struggle to bring NGS-based molecular profiling in house due to the associated costs and resources required, coupled with inconsistent payer coverage and challenges with reimbursement (Figure S3). Additionally, only 26% of the labs that rely on send-out NGS-based molecular profiling have a reflex protocol for NGS testing upon a patient’s metastatic diagnosis. However, most labs that offer patients in-house NGS-based molecular profiling today automatically reflex to NGS tests at the time of metastatic diagnosis.

### 3.2. Perceived Barriers and Future Outlook

Payers report placing restraints on NGS-based molecular profiling primarily due to the perception that clinicians do not order guideline concordant testing for patients diagnosed with metastatic breast cancer (Figure S4). Restrictions may come in several forms, such as the number of genes to be included on a panel (i.e., coverage of a small targeted panel or comprehensive genomic panels), the total number of tests patients may receive after metastatic diagnosis, the testing timepoint during the patient journey (i.e., at diagnosis or after progression), and the sample type (i.e., tissue- or liquid-biopsy based testing). Similarly, even if medical oncologists do order NGS-based molecular profiling for their patients, it may be restricted along these same lines and be sub-optimal or less comprehensive than guideline recommendations.

Further, HCPs may sub-optimally select what sample type to test for their patients diagnosed with metastatic breast cancer based on the incorrect assumption that liquid-based and tissue- based NGS test results are concordant. Nearly half of healthcare professionals report not being aware of the level of concordance between tissue-based and liquid-based NGS results (Figure S5). As a result, only a minority of HCPs consistently report following NCCN guidelines to consider reflexing to tissue-based NGS after a patient receives a negative liquid-based test at the time of mBC diagnosis or progression.

While all stakeholders claim to be aware of testing guidelines, 25% of medical oncologists and 33% of payers are unaware of NCCN guidelines to test patients diagnosed with metastatic breast cancer upon metastatic diagnosis (Table S1). However, most of these HCPs and payers plan to revise their clinical practice and coverage policies, respectively, based on the disclosure of new information.

**Figure 1:**
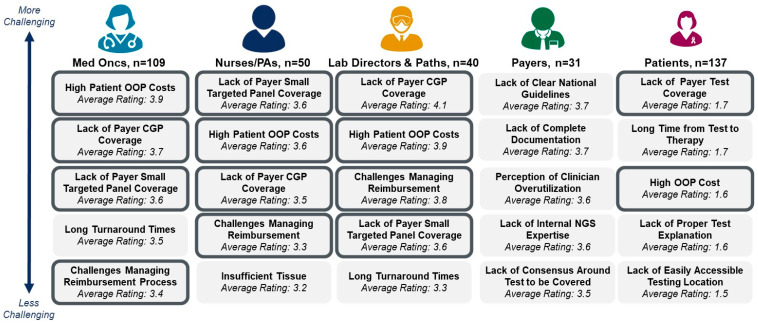
Perceived Barriers to NGS-based Molecular Profiling for Patients Diagnosed with HR+/HER2− Metastatic Breast Cancer. Respondents were asked to rate various barriers on a scale of 1 to 5, where 5 is very challenging and 1 is not challenging. The top 5 barriers for each stakeholder are reported. Barriers with a bold outline refer to barriers associated with reimbursement and coverage. OOP = out of pocket, CGP = comprehensive genomic profiling.

HCPs, lab directors and pathologists, and to a lesser extent patients, highlight payer policies—including test coverage, high out of pocket costs (OOP), and challenging reimbursement management—as the most significant barriers to accessing NGS-based molecular profiling for patients diagnosed with metastatic breast cancer (Figure 1). In contrast, payers highlight uncertainty around ideal NGS panel size, lack of clear national clinical guidelines and internal expertise on NGS-based molecular profiling as key challenges for broader test access to patients with mBC.

**Figure 2:**
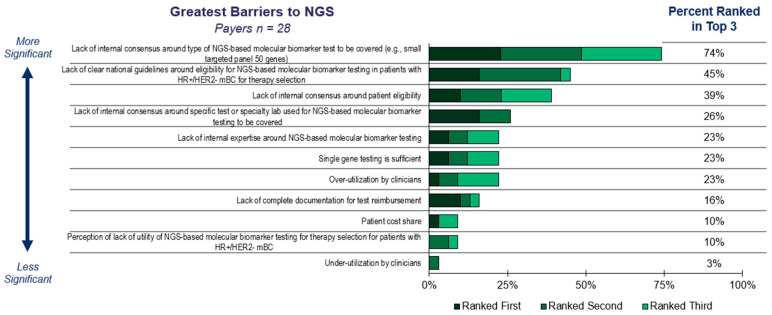
Payer’s Ranking of the Top Perceived Barriers to NGS-based Molecular Profiling for Patients Diagnosed with HR+/HER2− Metastatic Breast Cancer. Payers were asked to rank various barriers from 1 to 11. The percentage of payers that ranked each barrier as either their first, second, or third choice are plotted.

These barriers highlighted by payers (Figure 2) contribute to other downstream effects that lead to suboptimal NGS-based molecular profiling. According to our survey, only 55% of payers are covering NGS-based molecular profiling upon metastatic diagnosis, and 71% cover after first line therapy progression. Furthermore, 32% of payers reported they do not cover repeat testing. This limitation in coverage leads 40% of HCPs surveyed to only order one NGS-based molecular profiling test, often waiting until after first line therapy progression (Figure S6A). Payers primarily attribute their restrictions on repeat testing to a lack of clear guidelines on which patients qualify for repeat NGS-based molecular profiling, and the presumption that a single test is sufficient for patients with mBC (Figure S6B). Additionally, payers limit the size of NGS-based molecular profiling panels, with 81% reported covering small, targeted panels and 74% covering comprehensive genomic profiling, leaving a gap in patients without access to more comprehensive profiling.

Outside of challenges with payer policies, HCPs, lab directors, and pathologists perceive turnaround time (TAT) to be an additional yet lower barrier to optimal testing. HCPs indicate that the TATs of the NGS-based molecular profiling results may be too long for some patients with disease burden, necessitating urgent treatment decisions such as chemotherapy, even if NGS-based molecular profiling results could have provided an alternative therapy option. This sentiment on long TATs is perceived to affect a greater share of patients diagnosed with metastatic breast cancer when tissue testing is performed at a send-out lab compared to in-house (Figure S7).

All stakeholders have mixed opinions on which stakeholder holds greater responsibility for suboptimal NGS-based molecular profiling among eligible patients with metastatic breast cancer, although all acknowledge that no stakeholder is actively trying to hinder appropriate testing (Figure S8).

In the future, HCPs, lab directors, and pathologists anticipate an increase in NGS-based molecular profiling among patients diagnosed with metastatic breast cancer as a result of enhanced education around guidelines and patient eligibility, as well as an influx of newly approved targeted therapies relying on NGS-based molecular profiling (Figure S9). Similarly, payers highlight guideline recommendations and FDA companion diagnostic (CDx) approvals as the top influences on their NGS-based molecular profiling coverage policies.

## 4. Discussion

This multi-stakeholder study of medical oncologists, nurses, physician assistants, lab directors, pathologists, payers, and patients in the United States demonstrates that there is consensus to support broad adoption of NGS-based molecular profiling for patients diagnosed with metastatic breast cancer. While stakeholders recognize the value of comprehensive tumor profiling, gaps in education and awareness of updated guidelines hinder widespread use of NGS-based molecular profiling for these patients.

### 4.1. Perceptions of Inconsistent Payer Coverage

The survey results indicate that inconsistent payer coverage is perceived as the primary barrier to NGS-based molecular profiling across all patients diagnosed with metastatic breast cancer. HCPs, lab directors, and pathologists highlight restrictive coverage of NGS panels, high out-of-pocket cost for patients, and challenges managing reimbursement as the top-rated barriers to offering broad NGS-based molecular profiling today for patients diagnosed with metastatic breast cancer. Patients also highlight the high out-of-pocket costs as one of their major hurdles. In contrast, payers highlight lack of clear national guidelines, lack of internal consensus around what size panel to cover, and lack of internal expertise on NGS-based molecular profiling as key barriers to testing. The disconnect between stakeholders regarding the cause of suboptimal testing can be traced back to unfamiliarity with guidelines and companion diagnostics approvals.

A notable contributor to the underutilization of guideline-recommended testing is the limited awareness among payers regarding current clinical guidelines. This lack of familiarity can lead to assumptions that certain tests are not endorsed or covered, particularly when they observe high volumes of test orders from clinicians. As a result, payer skepticism may arise, with the perception that such testing is excessive or non-concordant with established standards. This disconnect underscores the need for improved communication and education between clinical stakeholders and payers to ensure alignment with respect to evidence-based practices and coverage decisions.

With the introduction of numerous targeted therapies in the past few years, anticipated approvals in the future, and FDA approvals of novel tests, payers have a significant volume of updates to monitor and incorporate into policies. Therefore, payers may benefit from additional support managing policies. Furthermore, payers also highlighted a perception of clinician overutilization and lack of complete documentation from HCPs as barriers to access for patients. As a result, payers place utilization management controls on NGS-based molecular profiling due to their impression that clinicians are not ordering guideline concordant testing.

HCPs, lab directors, and pathologists appropriately perceive payer pushback to be a challenge even for guideline-supported testing and approved tests, as payers acknowledge they grapple to manage developing policies for NGS-based molecular profiling for patients diagnosed with metastatic breast cancer without well-established internal expertise.

### 4.2. Requirement for Enhanced Guideline Education

Stakeholders need certainty that NGS-based molecular profiling will be covered for patients diagnosed with metastatic breast cancer to address the gap in testing today. Payers and HCPs both report that they are interested in aligning with NCCN guidelines, including testing at metastatic diagnosis and repeat testing upon a negative liquid-based NGS test on tissue samples. Education is crucial to raise guideline awareness and enhance access to NGS-based molecular profiling.

Payers rely upon guidelines such as NCCN and ASCO to guide coverage decisions for patients diagnosed with metastatic breast cancer but still require additional support to monitor updates to these guidelines (Figure S10). One way to limit the information gap is by outsourcing NGS-based molecular profiling advisory to laboratory benefit managers (LBMs). LBMs may have the knowledge and level of expertise required to make recommendations on novel, high-value diagnostics, particularly NGS-based molecular profiling. 52% percent of payers surveyed are contracting with an LBM, with 88% of them relying on their LBM for molecular testing guidance. Adoption of technology solutions that integrate or flag changes from the most up to date clinical guidelines into software to streamline policy creation may also benefit both payers and LBMs by remaining current.

HCPs could also be better informed on the complexity and nuance between different NGS-based molecular profiling methodologies, including the level of concordance between liquid-based and tissue-based results. Enhanced provider education on clinical guidelines and relevant literature will ensure that HCPs order the right test at the right time for their patient to offer the best chance at identifying biomarkers that could lead to targeted therapy selection. The pharmaceutical industry can also play a role here by funding, developing, and disseminating continuing medical education modules tied to guideline updates.

### 4.3. Future of NGS-Based Molecular Profiling in Metastatic Breast Cancer

In the future, stakeholders are optimistic about the opportunity of increased NGS-based molecular profiling in patients diagnosed with metastatic breast cancer. Stakeholders suggest that with numerous targeted therapy approvals on the horizon, as well as further refinement and enhanced education around clinical guidelines, more patients will be able to receive NGS-based molecular profiling as part of their metastatic breast cancer journey.

Health systems can further advance NGS-based molecular profiling adoption through development of institutional practices or protocols. Labs can institute reflex testing protocols based on clinical guidelines upon metastatic diagnosis, whether it be an in-house or send-out test. Additionally, as payers work to update policies, health systems can develop internal processes for managing payer pushback on NGS-based molecular profiling.

Formal multistakeholder governance structures with representatives from NCCN, major private and public payers, oncologists, patient advocates, and health economists could also help keep all stakeholders informed and aligned on guidelines and recommended clinical practice. Collectively, engaging with and educating all stakeholders on the importance of NGS testing for patients diagnosed with metastatic breast cancer and clinical guidelines will unlock optimal testing and improved outcomes.

### 4.4. Limitations

This study was subject to several limitations that may point to topics to be addressed in future research. Although the survey respondents were representative of the United States HCP, lab director, pathologist, payer, and patient populations, the sample size may limit the applicability of data to the entire population. The data was not statistically tested to quantify the differences between the stakeholders.

## 5. Conclusions

The findings of this study may help build support for tearing down existing barriers to NGS testing for eligible patients with metastatic breast cancer. Enhanced educational efforts across stakeholders in support of guideline-concordant NGS-based molecular profiling will allow for the realization of the full benefits of personalized medicine.

## Figures and Tables

**Table 1:** Survey respondent demographics are detailed by stakeholder type. Medical oncologists, nurses, and physician assistants may be collectively referred to as “healthcare professionals” (HCPs) in the discussion.

**Medical Oncologists (*n* = 109)**		
	Average Number of Patients/Month	Average Molecular Biomarker Testing Rate
HR+/HER2− Metastatic Breast Cancer	59	77%
		Respondents
Geographic Region	West	20%
	Midwest	22%
	South	32%
	Northeast	26%
Practice Type	Private Practice	43%
	Academic Health System	34%
	Community Health System	23%
**Nurses and Physician Assistants (*n* = 50)**		
	Average Number of Patients/Month	Average Molecular Biomarker Testing Rate
HR+/HER2− Metastatic Breast Cancer	37	66%
		Respondents
Geographic Region	West	12%
	Midwest	32%
	South	28%
	Northeast	28%
Practice Type	Private Practice	42%
	Academic Health System	34%
	Community Health System	24%
**Lab Directors and Pathologists (*n* = 40)**		
	Average Number of Specimens/Month	NGS-Based Biomarker Testing Rate
Breast Cancer	85	40%
		Respondents
Geographic Region	West	23%
	Midwest	15%
	South	35%
	Northeast	28%
Practice Type	Independent Reference Lab	15%
	Academic Health System	53%
	Community Health System	32%
**Payers (*n* = 31, 52% Commercial, 48% CMS)**		
		Respondents
Geographic Region	West	29%
	Midwest	23%
	South	29%
	Northeast	19%
Average Plan Size (Lives)	10,000–100,000	23%
	100,000–1,000,000	23%
	1,000,000–5,000,000	26%
	5,000,000–10,000,000	19%
	>10,000,000	10%
**mBC Patients (*n* = 137)**		
		Respondents
Sex	Female	100%
Geographic Region	West	16%
	Midwest	26%
	South	35%
	Northeast	23%
Ethnicity	White	77%
	Latin American/Hispanic	10%
	Black/African American	10%
	American Indian or Alaska Native	3%
	Asian	1%
	Prefer Not to Say	2%
Insurance Type	Commercial Insurance	50%
	Medicare	28%
	Medicaid	13%
	Medicare and Medicaid	7%
	Other	2%

## Data Availability

The data presented in this study are available on reasonable request from the corresponding author.

## Supplementary Materials

The following supporting information can be downloaded at https://www.mdpi.com/article/10.3390/diagnostics15202626/s1: Figure S1: NGS-based Molecular Profiling Testing Paradigm in mBC; Figure S2: NGS Testing Drivers; Figure S3: Barriers to In-House NGS Testing; Figure S4: Rationale for Restricting Coverage on NGS-based Molecular Profiling; Figure S5: Perceived Concordance between Tissue-based and Liquid-based NGS Test Results; Figure S6: Perceived Barriers to Offering Multiple NGS-based Molecular Profiling Tests to Patients with mBC; Figure S7: Patient Treatment Impact of Long Turnaround Time; Figure S8: Stakeholder Impact on NGS Test Access; Figure S9: Rationale for Future Uptake on NGS Testing—Stakeholders’ Perspectives; Figure S10: Educational Resources Utilized—Stakeholders’ Perspectives; Table S1: Awareness and Willingness to Change with NCCN Guideline Knowledge.
