# Supplementary material for: Perceived Barriers to NGS-Based Molecular Profiling Among US Metastatic Breast Cancer Patients
Source: Diagnostics (Basel). 2025 Oct 17;15(20):2626. doi: 10.3390/diagnostics15202626 (PMC12564138; doi:10.3390/diagnostics15202626)
Supplement: Supplementary file 1 [file diagnostics-15-02626-s001.zip › diagnostics-3796930-supplementary.pdf]

Perceived Barriers to NGS-Based Molecular Profiling among US Metastatic Breast Cancer Patients

Supplementary Figures and Tables

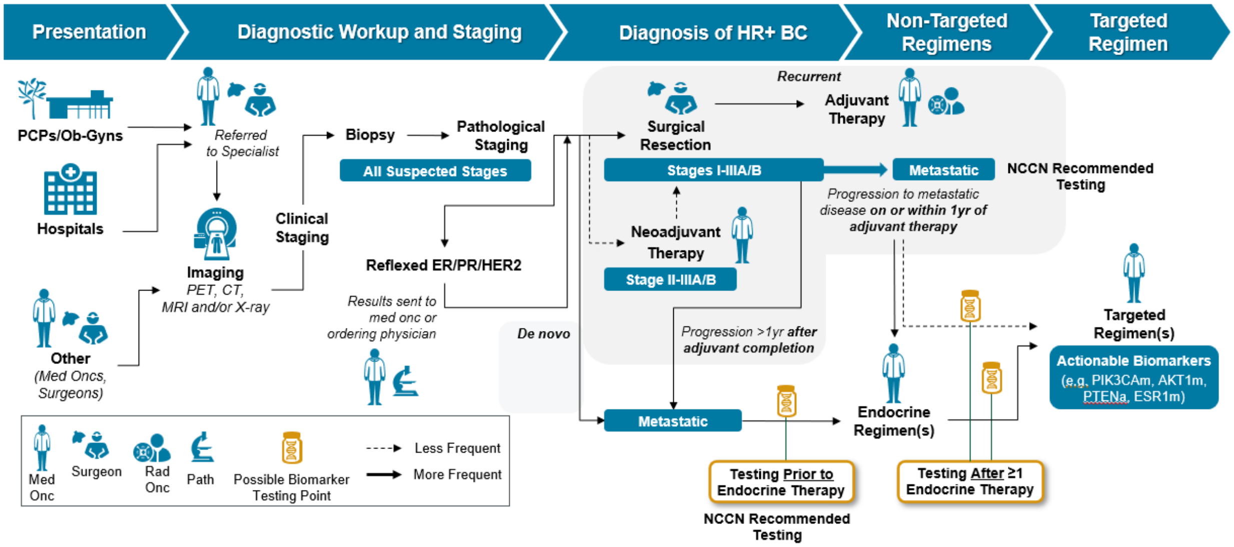

Figure S1: NGS-based Molecular Profiling Testing Paradigm in mBC

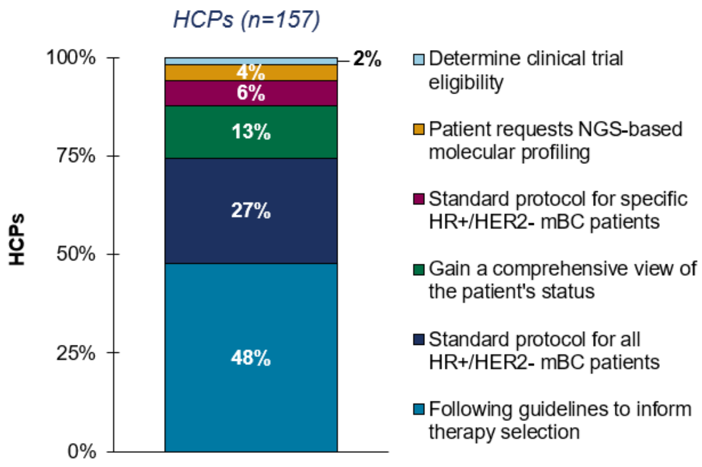

Figure S2: NGS Testing Drivers. Percentages depict the share of HCP respondents that selected each response as the primary reason for using NGS-based molecular profiling for patients.

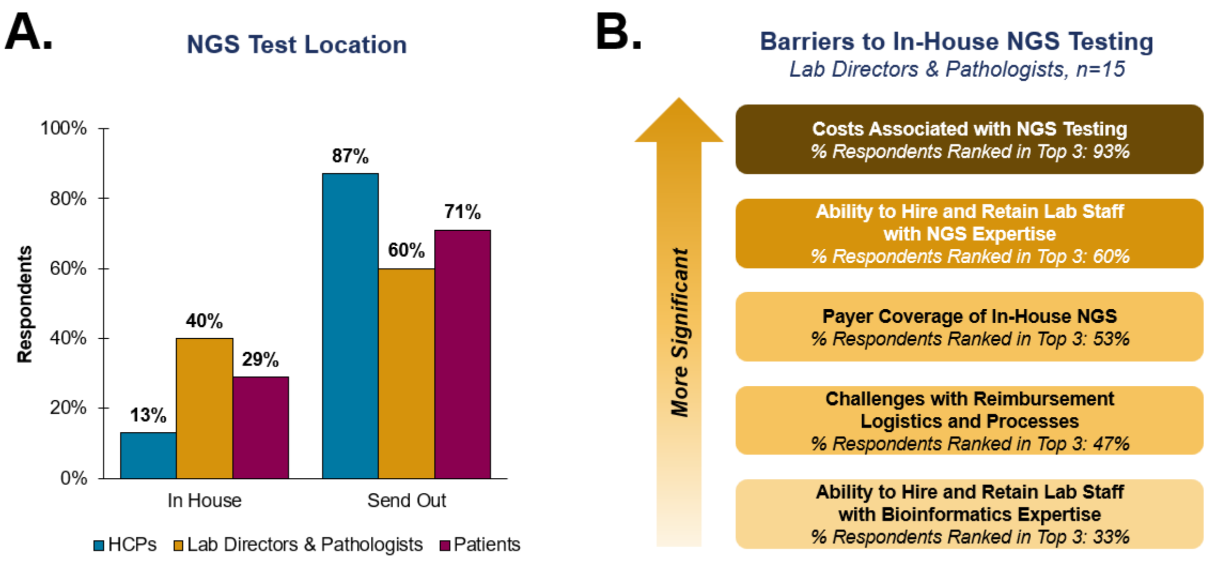

Figure S3: Barriers to In-House NGS Testing A. Respondents depict where NGS-based molecular profiling is most commonly performed for clinical practice. **B.** Lab directors rank which barriers are most significant to bringing NGS testing in-house. 79% of lab directors and pathologists who do not currently perform NGS testing in-house do not plan to bring NGS testing in-house.

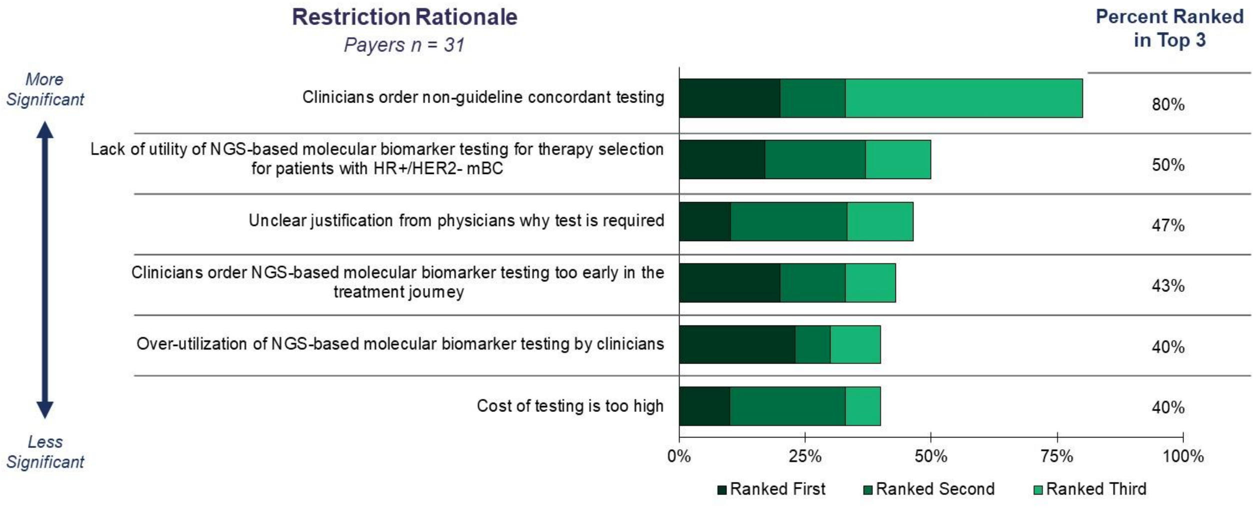

Figure S4: Rationale for Restricting Coverage on NGS-based Molecular Profiling.

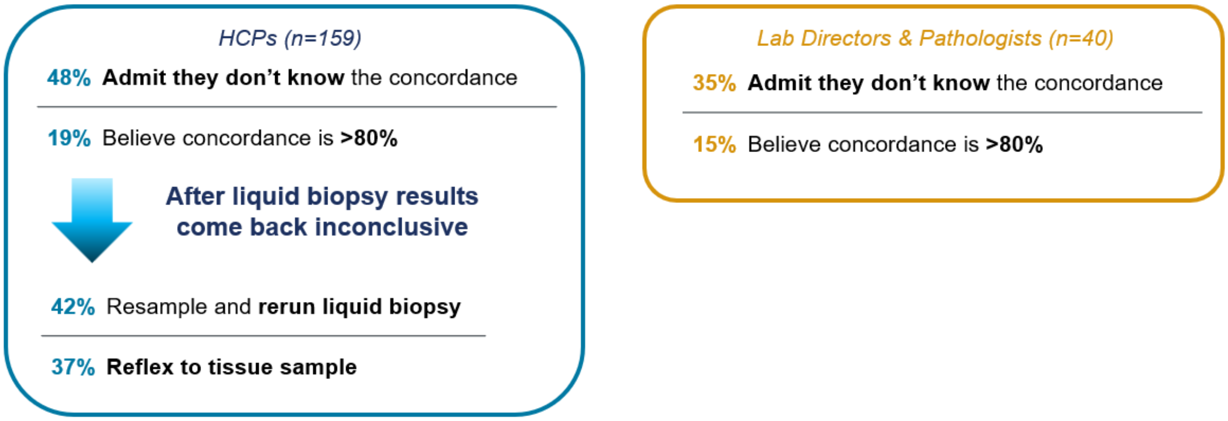

Figure S5: Perceived Concordance between Tissue-Based and Liquid-Based NGS Test Results.

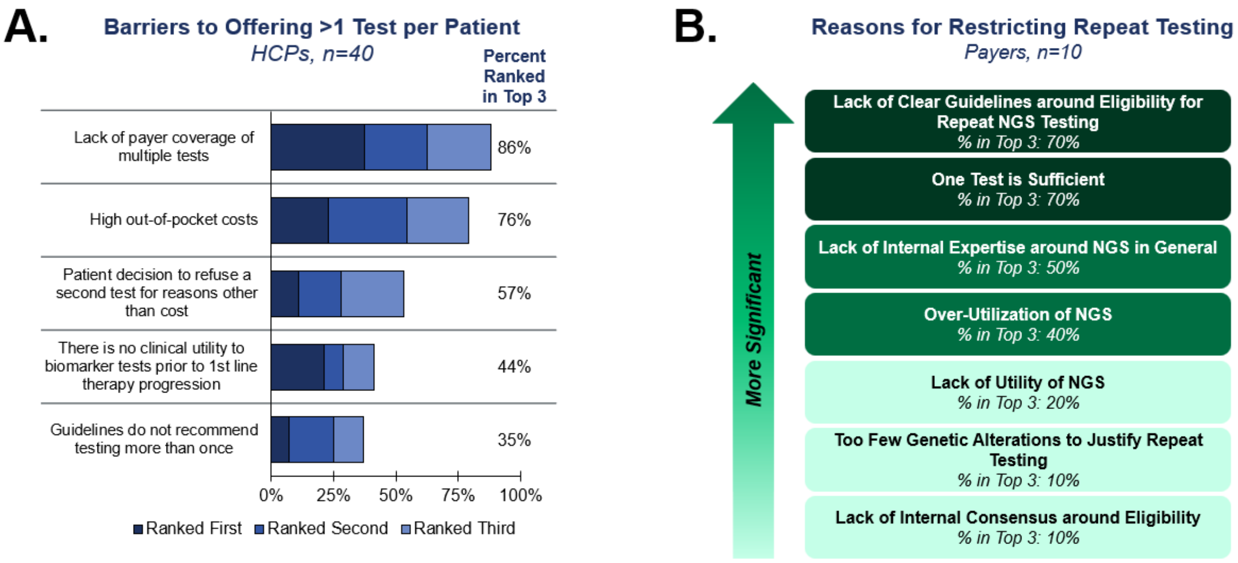

Figure S6: Perceived Barriers to Offering Multiple NGS-based Molecular Profiling Tests to Patients with mBC. A. HCP's perspective **B.** Payer's perspective**.**

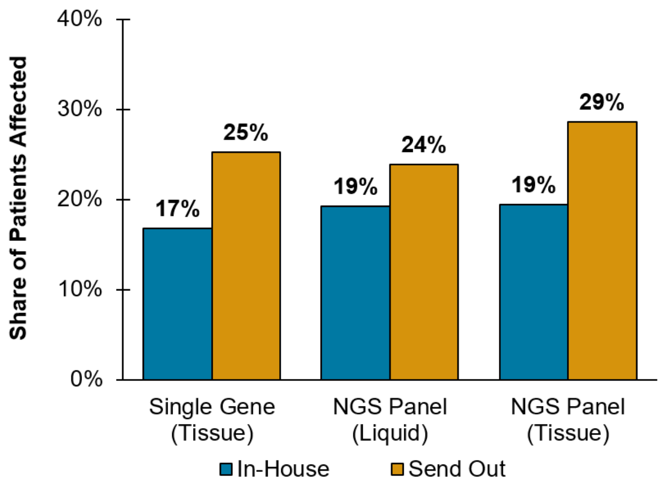

Figure S7: Patient Treatment Impact of Long Turnaround Time. HCPs depict the share of patients for which the turnaround time for testing results to determine a targeted therapy is sub-optimal and instead must initiate chemotherapy.

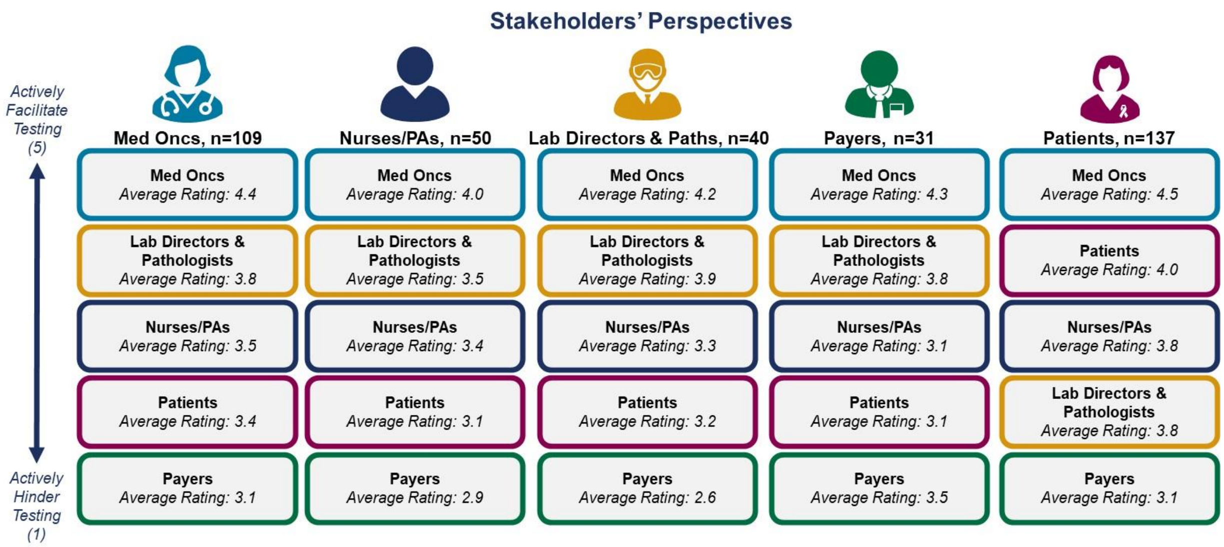

Figure S8: Stakeholder Impact on NGS Test Access. Each stakeholder rated the level of impact of different stakeholders on the utilization of NGS-based molecular profiling on a scale of 1 to 5 where a 1 rating indicates the stakeholder actively hinders testing and a 5 rating indicates the stakeholder actively facilitates testing.

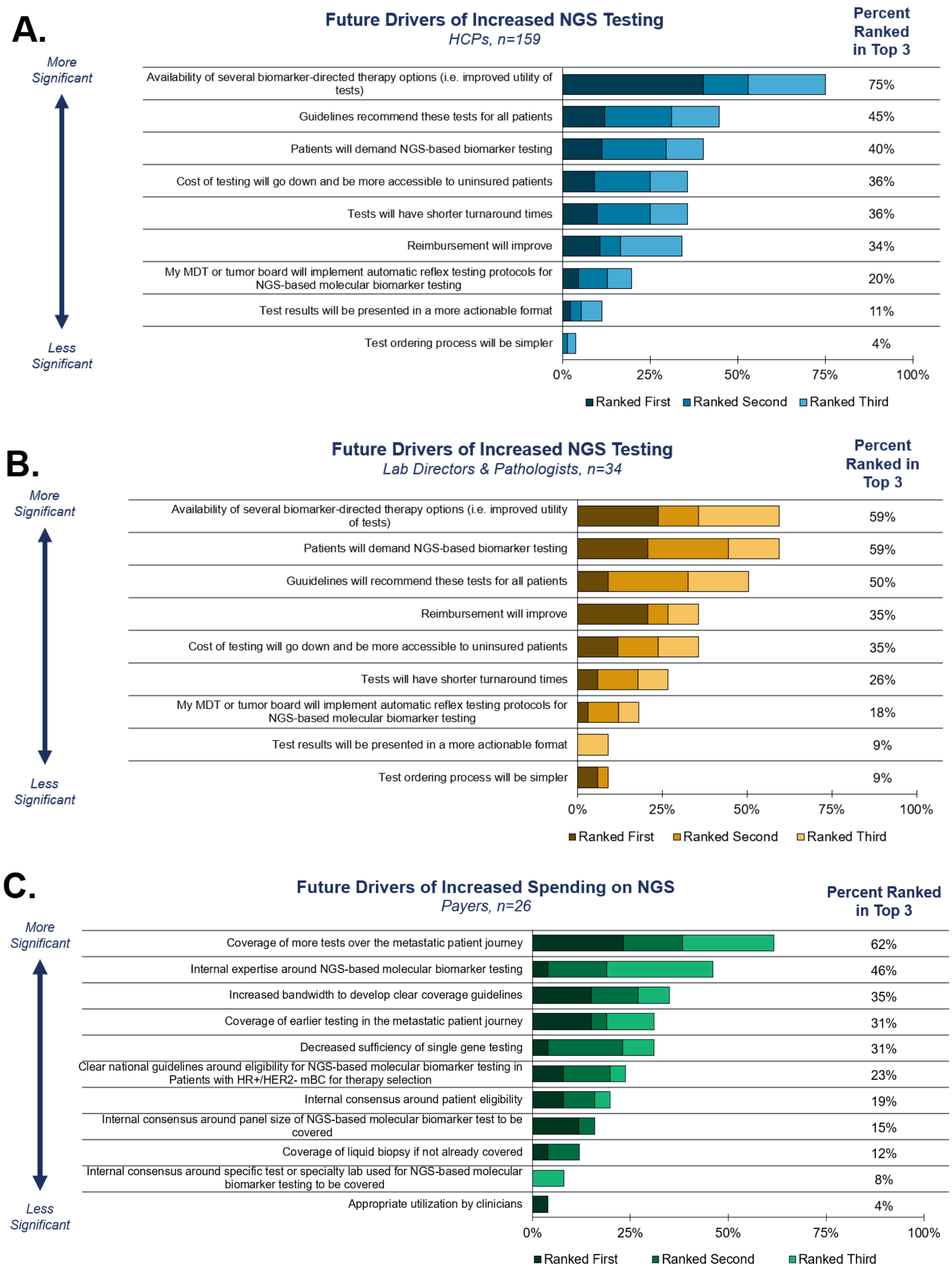

Figure S9: Rationale for Future Uptake on NGS Testing: Stakeholders' Perspectives. A. HCPs B. Lab Directors & Pathologists C. Payers.

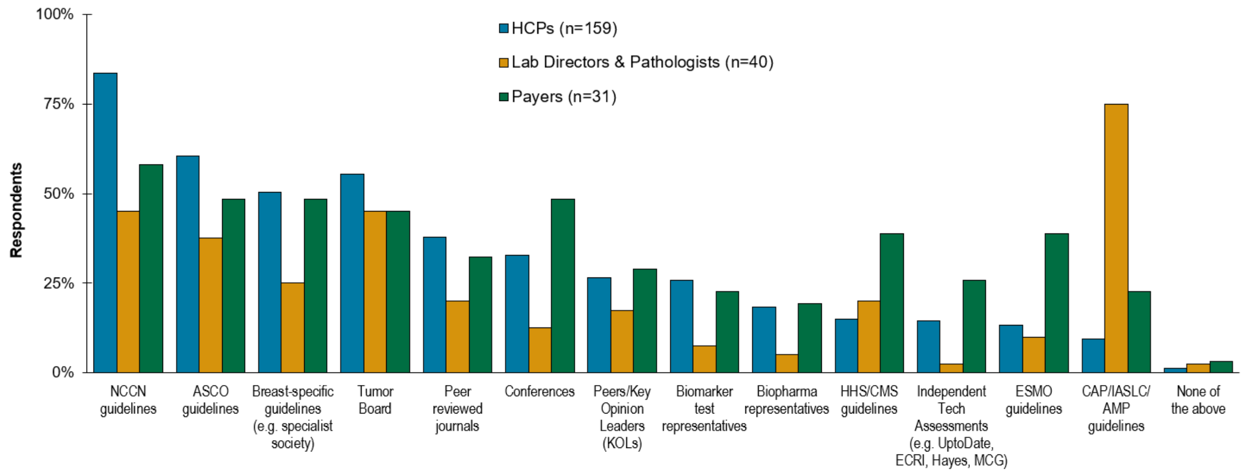

Figure S10: Educational Resources Utilized: Stakeholders' Perspectives.

Table S1: Awareness and Willingness to Change with NCCN Guideline Knowledge

Stakeholders	Lack of awareness of NCCN's recommendation on comprehensive germline and somatic profiling at mBC diagnosis	Intention to update testing behavior and coverage upon education
Medical Oncologist	25%	59% (of the 25%)
Payer	33%	65% (of the 33%)
